# A novel point-of-care oral anti-HCV assay: Is it reliable for screening hepatitis C virus infection in the era of direct-acting antivirals?

**DOI:** 10.1371/journal.pone.0211795

**Published:** 2019-02-12

**Authors:** Rui-Feng Yang, Yan Liu, Cai-Yan Zhao, Ya-Xing Ding, Yu Chen, Ya-Dong Wang, Zhong-Ping Duan

**Affiliations:** 1 Peking University People’s Hospital, Peking University Hepatology Institute, Beijing Key Laboratory of Hepatitis C and Immunotherapy for Liver Diseases, Beijing, China; 2 Infectious Diseases Department, the Third Hospital of Hebei Medical University, Shijiazhuang, China; 3 Tianjin Center for Diseases Control and Prevention, Tianjin, China; 4 Artificial Liver Center, Beijing You’an Hospital, Capital Medical University, Beijing, China; Kaohsiung Medical University Chung Ho Memorial Hospital, TAIWAN

## Abstract

Recent advance in the direct-acting antivirals (DAAs) offers the potentials to eradicate hepatitis C virus (HCV) worldwide and makes universal screening more urgent. A point-of-care (POC) oral anti-HCV assay, the Fortune assay, was developed and its performance was evaluated. Individuals with or without HCV infection were recruited in three Centers. Paired oral and serum samples were tested using the Fortune and InTec anti-HCV assays. The Kehua serum anti-HCV assay served as a supplemental test to verify the discordant results. Some oral samples were also tested using the OraQuick anti-HCV assay. Furthermore, the Fortune assay results were compared with the documented RNA results. Sensitivity, specificity, and accuracy of the Fortune assay was 93.11%, 98.48%, and 96.58%, respectively (n = 1,022). Consistency between the Fortune and OraQuick assays was 96.35% (264/274); the Fortune assay detected additional 8 positive oral samples missed by the OraQuick assay. The Fortune assay demonstrated a 97.46% (115/118) positivity among the viremic patients. Furthermore, its sensitivity was HCV genotype independent. In conclusion, the Fortune assay was highly specific and accurate. It had comparable sensitivity as the serum assays for the diagnosis of active HCV infection. It provides a completely non-invasive and reliable tool for HCV screening in the DAA era.

## Introduction

Hepatitis C virus (HCV) affects 115 million people worldwide (i.e. 1.6% global anti-HCV seroprevalence)[1], and the viremic (HCV RNA positive) prevalence is estimated to be 1.1%. HCV infection is more prevalent in special populations such as intravenous drug users (IDUs), hemodialysis patients, cancer patients, and paid blood donors [2]. Chronic HCV infection (CHC) is the major cause of liver cirrhosis and hepatocellular carcinoma in the Western countries. In many other countries where the HCV receives little attention, however, the disease burden is much higher [3]. In recent years, with the revolutionary development of the direct-acting antivirals (DAAs), 95%-100% of patients can achieve sustained virological response (SVR) after 8 to 12 weeks of oral administration [4]. Most of the patients ineligible or intolerant for the treatment with pegylated interferon (PEG-IFN) α plus ribavirin can also be cured using DAAs. It is more urgently needed that more patients be diagnosed and linked to timely treatment to reduce the disease burden in the era of DAAs than in the past [5]. On the other hand, as a “silent killer”, HCV infection is often asymptomatic, and many infectors, including the university hospital health care providers, are unware of their status until they have abnormal liver tests or develop the symptoms of cirrhosis [6–8]. In China, there are approximately 10 million of HCV infected patients, while only 2% are registered in the National epidemic prevention and control network platform annually [9, 10]. Achievement of the global HCV elimination first requires effective screening programs, including risk-based screening, general population screening and birth cohort screening programs [7, 11]. Unfortunately, there has been a lack of screening programs in most developing countries [12].

The screening and diagnosis of HCV infection relies heavily on the laboratory assays, among which serum anti-HCV testing is the first of choice [13]. Nevertheless, under traditional cultural or special historical atmosphere, or in poor medical conditions, many Chinese people, especially those from the resource-limited areas, or those with high risk of infection due to previous unregulated plasmapheresis[14], are reluctant, or have no access to submit their blood samples for screening. As we know, serum contents such as drugs, antigens and antibodies can be transferred to oral fluid by passing through capillary walls in salivary gland tissues [15]. Antibodies can be detected in the oral fluid as well [16]. Lee et al.[17] found that the sample types (whole blood, serum or plasma, and oral fluid) had little influence on the anti-HCV detection results. Therefore, oral assays might help clear the HCV screening barrier [7, 18]. It is also suitable for the IDUs with poor vein access. Recently, a novel point-of-care (POC) oral anti-HCV assay, the Fortune anti-HCV assay, has been developed. It is a non-invasive and non-instrumental assay, facilitating the rapid screening of HCV infection. Its performance was evaluated in a large Chinese population from three Centers.

## Materials and methods

### Subjects

The study was conducted at the Department of hepatology or infectious diseases of three Centers, the Capital Medical University Beijing You’an Hospital (Center 01), Peking University People’s Hospital (Center 02) and the Third Hospital of Hebei Medical University (Center 03). Either the inpatient or outpatient with or without HCV infection was enrolled. Apparently healthy subjects seeking for virological tests or vaccination were enrolled as well. Primary diagnosis was made according to the patient’s medical history and the laboratory tests. Diagnosis the HCV infection met the criteria provided by the Guideline of prevention and treatment for hepatitis C [10]. The study was performed with approval from the institutional review board of each center. All patients provided written informed consent before the oral and/or blood sample collection.

### Oral fluid and serum collection

**Fig 1:**
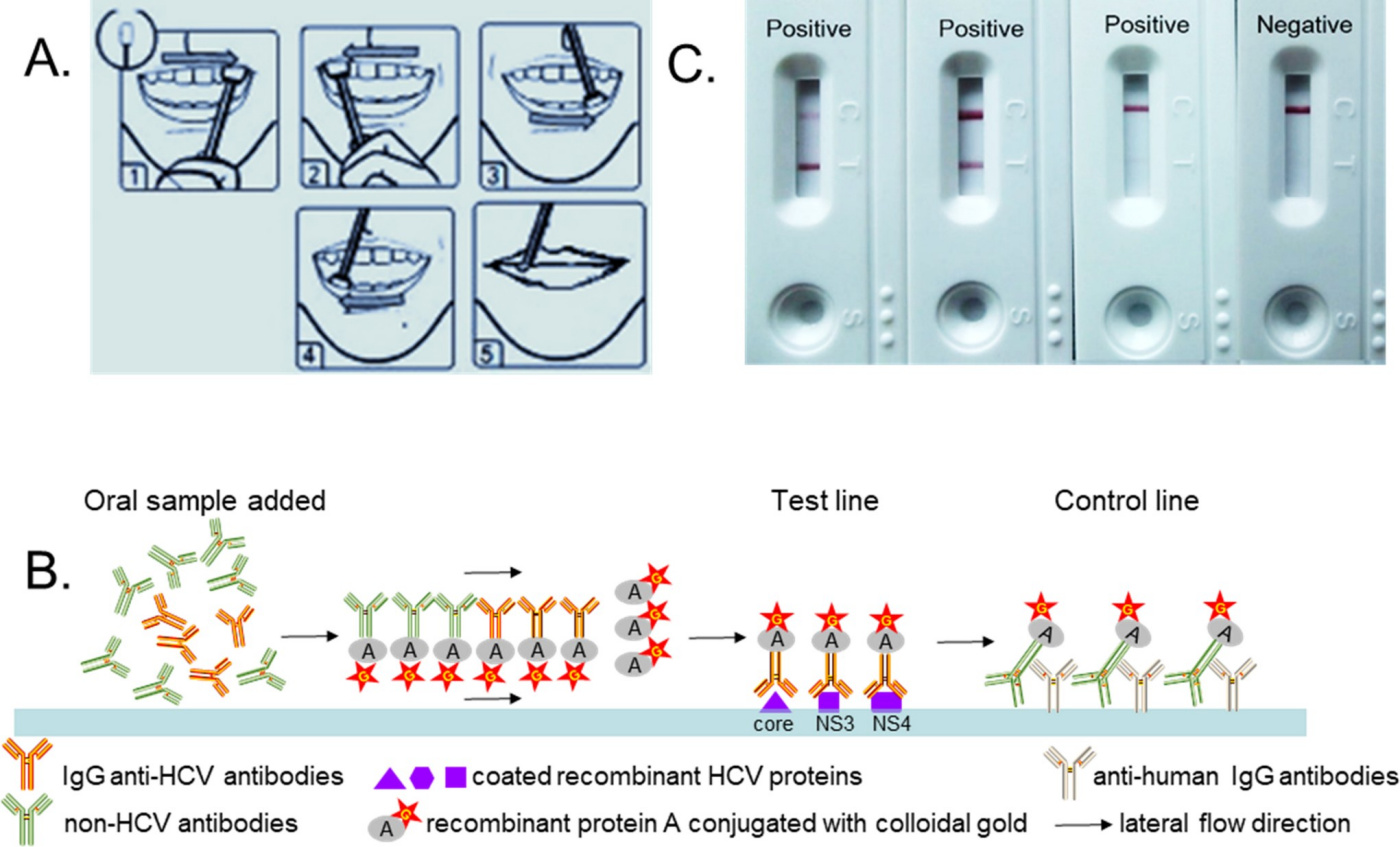
Sample collection (A.), schematic diagram of the immunoreaction (B.), and result interpretation (C.) of the Fortune point-of-care oral anti-HCV assay.

To collect oral fluid sample, place the swab between the subject’s buccal mucosa and gingiva, and move it back and forth for 5 times. Then keep the swab in mouth for 2 minutes to make it soaked with oral fluid. Thereafter, immerse the swab into the dilution buffer and release the oral fluid by squeezing it (Fig 1A). Venous blood should be collected alongside with the oral fluid. Nevertheless, if the paired serum sample with a leftover volume >0.5mL could be retrieved from the Department of clinical laboratory, where it was originally collected for biochemical or virological assays within the recent week, venipuncture could be avoided. The serum and oral fluid samples were kept in -20°C until further analysis.

### Assays

#### The Fortune point-of-care oral anti-HCV assay

The Fortune anti-HCV assay (Fortune Bioscience Co., Ltd, Zhenzhou, China) is an indirect immunoassay based on the colloidal gold technique (Fig 1B). The recombinant core, non-structural (NS) 3 and NS4 proteins (Polaris-Bio Co., Ltd, Beijing, China) are coated at the test line to capture the oral anti-HCV antibodies. A total of 100 μL of oral fluid is added. After 10 to 15 minutes of lateral flow, a visible reddish-purple test line, no matter how weak it is, indicates a positive result (Fig 1C). The control line captures non-HCV specific IgG antibodies and monitors the lateral flow and color development process; a result without the colored control line is indeterminate and the test should be repeated.

#### Serum anti-HCV assays

The InTec serum anti-HCV assay (InTec Products, Inc., Xiamen, China), an indirect immunoassay based on the colloidal gold technique, was used as the preferred reference assay. For the paired saliva and serum samples with discordant anti-HCV results, the serum was then subjected to the Kehua enzyme-linked immunosorbent assay (ELISA) (Kehua Bio-engineering Co., Ltd, Shanghai, China), which served as a supplemental test. A result with a signal-to-cut-off ratio (S/Co) ≥1.00 was positive.

#### OraQuick oral anti-HCV assay

Saliva samples with adequate volume were also tested using the OraQuick rapid anti-HCV assay (OraSure Technologies, Inc., Bethlehem, PA). It is CE marked and has the similar features as the Fortune assay.

#### HCV RNA assays

The clinical sensitivity of the Fortune oral anti-HCV assay for diagnosing active HCV infection (chronic or acute HCV infection) was also assessed. HCV RNA results within the most recent month were retrieved from the laboratory information systems (LIS) in the Centers. HCV RNA assays routinely used in each Center included the careHCV assay 3.0 (Qiagen Co., Ltd., Shanghai, China) with a limit of detection (LOD) of 100 IU/ml, the Sansure HCV RNA assay (Sansure Biotech Inc., Changsha, China) with a LOD of 50 IU/mL, and the Cobas Taqman HCV 2.0 assay (Roche Molecular Systems, Branchburg, NJ) with a LOD of 15 IU/mL. The proportion of the subjects with positive Fortune anti-HCV results in those with positive HCV RNA results was calculated.

#### HCV genotyping assays

Samples with confirmed positive anti-HCV results were first genotyped using an in-house assay based on polymerase chain reaction and Sanger sequencing [19, 20]. Briefly, the core sequence was amplified. Subsequently, the phylogenetic tree was constructed on the MEGA 6.05 software. GenBank accession numbers of the reference sequences of genotypes 1–7 were AF009606 (1a), D90208 (1b), D00944 (2a), D17763 (3a), D49374 (3b), Y11604 (4a), Y13184 (5a), Y12083 (6a), D84262 (6b), and EF108306 (7a). This molecular genotyping assay provided genotype and subtype results. In the meantime, the amino acid sequences of different genotypes were compared as well.

For the remaining samples which could not be genotyped, a serotyping assay was conducted [21]. It identified genotypes 1 and 2 as well as non-1/2 genotype. The assay provided genotype results but could not identify subtypes.

### Experimental procedures

All the experiment procedures were performed in accordance with the National regularities and the manufacturers’ instructions. Oral fluid and serum samples were tested separately by different technicians, and the results were interpreted independently in order to minimize bias. HCV genotyping tests were performed at Peking University Hepatology Institute. Results were pooled and statistically analyzed.

### Statistics

Sensitivity, specificity, accuracy, positive predictive value (PPV), negative predictive value (NPV) and their 95% confidence intervals (CI) were calculated. Kappa value was used to assess the extent of consistency; Kappa value >0.75 indicates a high consistency, and <0.4 a weak consistency. Sensitivity of the Fortune assay among different genotypes was compared using the Fisher’s exact test. Statistics were performed using the GraphPad Prism 6.02 software (GraphPad Software Inc., San Diego, CA). Statistical significance is defined as P<0.05.

## Results

### Information of the subjects

A total of 1022 subjects were enrolled, including 546 males and 476 females with the average age of 46.3 years. The information is listed in Table 1.

**Table 1:** Clinical features of the study population.

Primary diagnosis		Center			Total
		01	02	03	
**HCV infection**		154	78	136	368
	acute hepatitis C	1	1	0	2
	chronic hepatitis C (active or resolved)	147	74	124	345
	HCV related liver cirrhosis	5	2	12	19
	HCV related hepatocellular carcinoma (HCC)	0	1	0	1
	HBV co-infection	1	0	0	1
**Non-HCV-related liver diseases**		148	100	268	516
	Hepatitis B virus (HBV) surface antigen positive (HBV carrier status, chronic hepatitis B, and HBV related liver cirrhosis or HCC)	67	31	161	259
	non-alcoholic fatty liver disease	12	25	17	54
	alcoholic liver disease	9	2	17	28
	drug-induced liver injury	24	24	10	58
	autoimmune liver disease (autoimmune hepatitis and primary biliary cirrhosis)	17	3	33	53
	Hepatitis A virus IgM positive	2	4	9	15
	Hepatitis E virus IgM positive	4	9	0	13
	alanine aminotransferase elevation without exact cause	13	2	21	36
**Diseases of other organs**		0	20	26	46
	preoperative screening	0	20	24	44
	fever	0	0	1	1
	diarrhea	0	0	1	1
**Apparently healthy**		51	11	15	77
**Not diagnosed due to limited data**		0	0	15	15
**Total**		353	209	460	1022

### Consistency of the Fortune oral anti-HCV results and the serum anti-HCV results

A total of 1022 paired oral and serum samples were tested in parallel using the Fortune oral assay and the InTec serum assay. There were 37 pairs of samples with inconsistent results. As the Kehua serum assay was used as a supplementary test, 35 samples yielded consistent results as with the InTec assay, and the remaining 2 samples yield consistent results as with the Fortune assay. The data are shown in Table 2.

**Table 2:** Performance characteristics of the Fortune oral anti-HCV assay.

Oral anti-HCV (Fortune assay)	Serum anti-HCV (first the InTec assay, and Kehua assay as a reflex test)								
	Center 01		Center 02		Center 03		Centers 01–03		
	Positive	Negative	Positive	Negative	Positive	Negative	Positive	Negative	Total
**Positive**	138	4	71	1	129	5	338	10	348
**Negative**	16	195	2	135	7	319	25	649	674
**Subtotal**	154	199	73	136	136	324	363	659	1022
**Sensitivity (%) (95% CI)**	89.61 (83.68, 93.94)		97.26 (90.45, 99.67)		94.85 (89.68, 97.91)		93.11 (90.00, 95.49)		
**Specificity (%) (95% CI)**	97.99 (94.93, 99.45)		99.26 (95.97, 99.98)		98.46 (96.44, 99.50)		98.48 (97.23, 99.27)		
**Accuracy (%) (95% CI)**	94.33 (91.92, 96.75)		98.56 (96.95, 100)		97.39 (95.93, 98.85)		96.58 (95.46, 97.69)		
**PPV (%) (95% CI)**	97.18 (92.94, 99.23)		98.61 (92.50, 99.96)		96.27 (91.51, 98.78)		97.13 (94.48, 98.61)		
**NPV (%) (95% CI)**	92.42 (87.98, 95.60)		98.54 (94.83, 99.82)		97.85 (95.63, 99.13)		96.29 (94.57, 97.59)		
**Kappa value**	0.88		0.97		0.94		0.93		

CI, confidence interval; PPV, positive predictive value; NPV, negative predictive value

### Comparison of the oral anti-HCV antibody results released by the Fortune and OraQuick assays

There were 274 oral samples available for the OraQuick assay. The anti-HCV results by the two oral assays showed a consistency of 96.35% with a Kappa value of 0.93 (Table 3). Ten oral samples gave inconsistent results (Table 4).

**Table 3:** Comparison of the anti-HCV results using the Fortune and OraQuick oral assays.

Fortune assay	OraQuick assay		Total
	Positive	Negative	
**Positive**	97	9	106
**Negative**	1	167	168
**Total**	98	176	274

**Table 4:** Oral fluid samples with discordant anti-HCV results released by the Fortune and OraQuick assays (n = 10).

Subject ID	OraQuick assay	Fortune assay	Serum anti-HCV result	HCV RNA (IU/mL)	Antiviral treatment
**10087**	-	+	+	NA	NA
**10111**	-	+	+	1.01×10^7^	treatment naive
**10126**	-	+	+	-	sofosbuvir/ledipasvir, SVR
**10143**	-	+	+	-	sofosbuvir monotherapy, SVR
**20031**	-	+	+	-	PEG-IFN and ribavirin, SVR
**20044**	+	-	+	-	sofosbuvir/daclatasvir, treated for 11 weeks
**20060**	-	+	+	5.24×10^5^	treatment naive
**30006**	-	+	-	not tested	no therapeutic indication
**30089**	-	+	+	-	PEG-IFN and ribavirin, SVR
**30118**	-	+	+	-	sofosbuvir/daclatasvir, SVR

+, positive; -, negative; NA, data not available; PEG-IFN, pegylated interferon alpha; SVR, sustained virological response

### Sensitivity of the Fortune assay for detecting samples of the subjects with archived positive HCV RNA results

The HCV RNA results of 285 subjects could be traced from the LIS within the past six months. A total of 118 subjects had positive HCV RNA results, among which 115 gave positive oral anti-HCV results by the Fortune assay, and the remaining 3 samples had negative results (Table 5). The 3 subjects were all from Center 01, with high viral load (>2×10^5^ IU/mL). Two of them also had high-level serum anti-HCV (S/Co >10.0 by the Kehua ELISA). The remaining one had a negative serum anti-HCV result by the InTec assay, and hence the serum sample was not further tested using the Kehua assay.

**Table 5:** The results released by the Fortune oral anti-HCV assay in patients with documented positive HCV RNA results.

Oral anti-HCV (Fortune assay)	Archived positive HCV RNA results						
	Center 01		Center 02		Center 03		Total
	Sansure HCV RNA	Taqman HCV 2.0	careHCV 3.0	Taqman HCV 2.0	Sansure HCV RNA	Taqman HCV 2.0	
**Positive**	45	14	6	32	18	0	115
**Negative**	2	1	0	0	0	0	3
**Subtotal**	47	15	6	32	18	0	118
**Sensitivity (%)**	95.74	93.33	100.00	100.00	100.00	-	97.46

### Sensitivity of the Fortune assay among different viral genotypes

**Fig 2:**
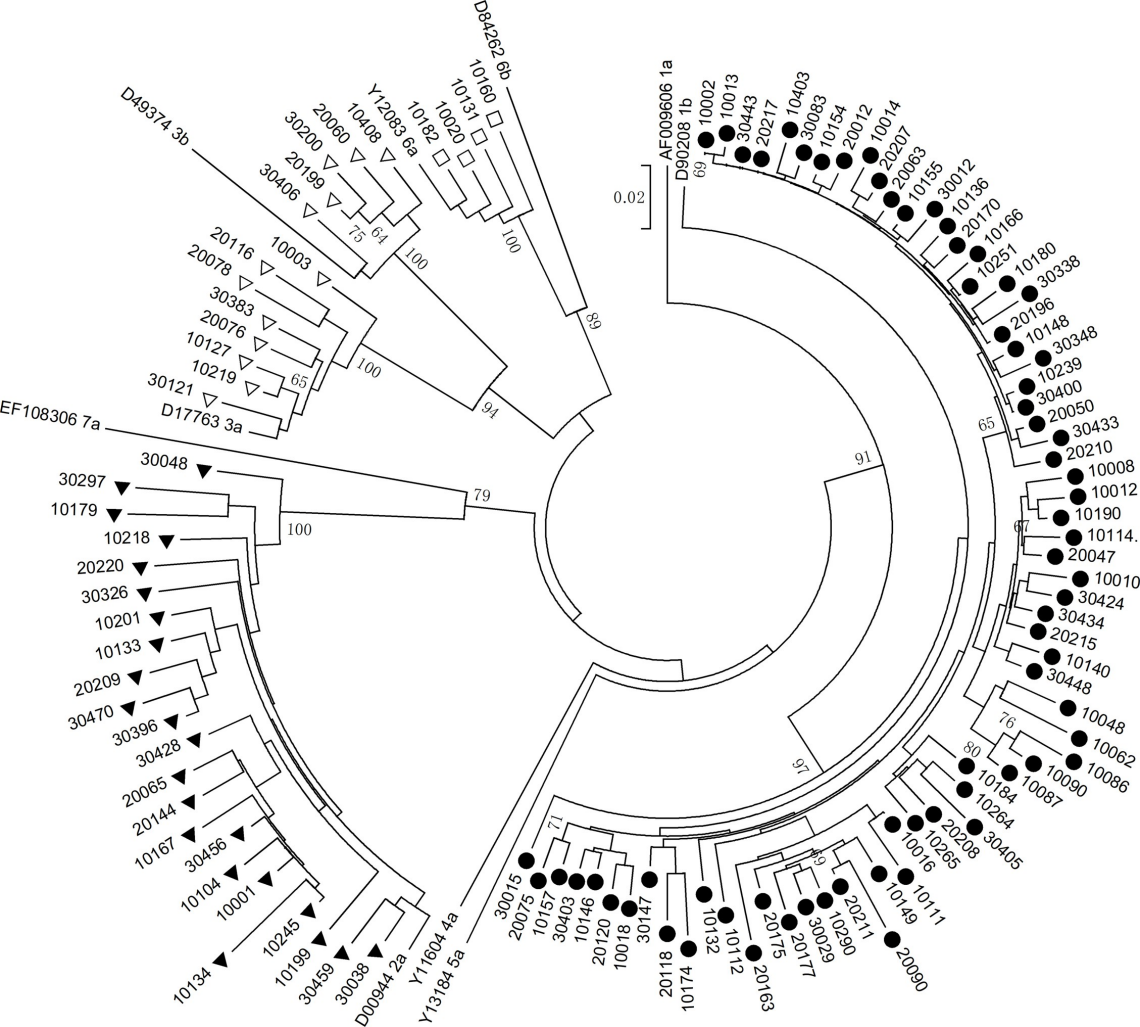
Phylogenetic trees of the core sequences of 110 HCV strains with genotypes 1, 2, 3 and 6. Bootstrap percentages are shown at the node of each main branch. The bar indicates the genetic divergence. ●, genotype 1b; ▲, genotype 2a; △genotype 3a or 3b; □, genotype 6a.

**Fig 3:**
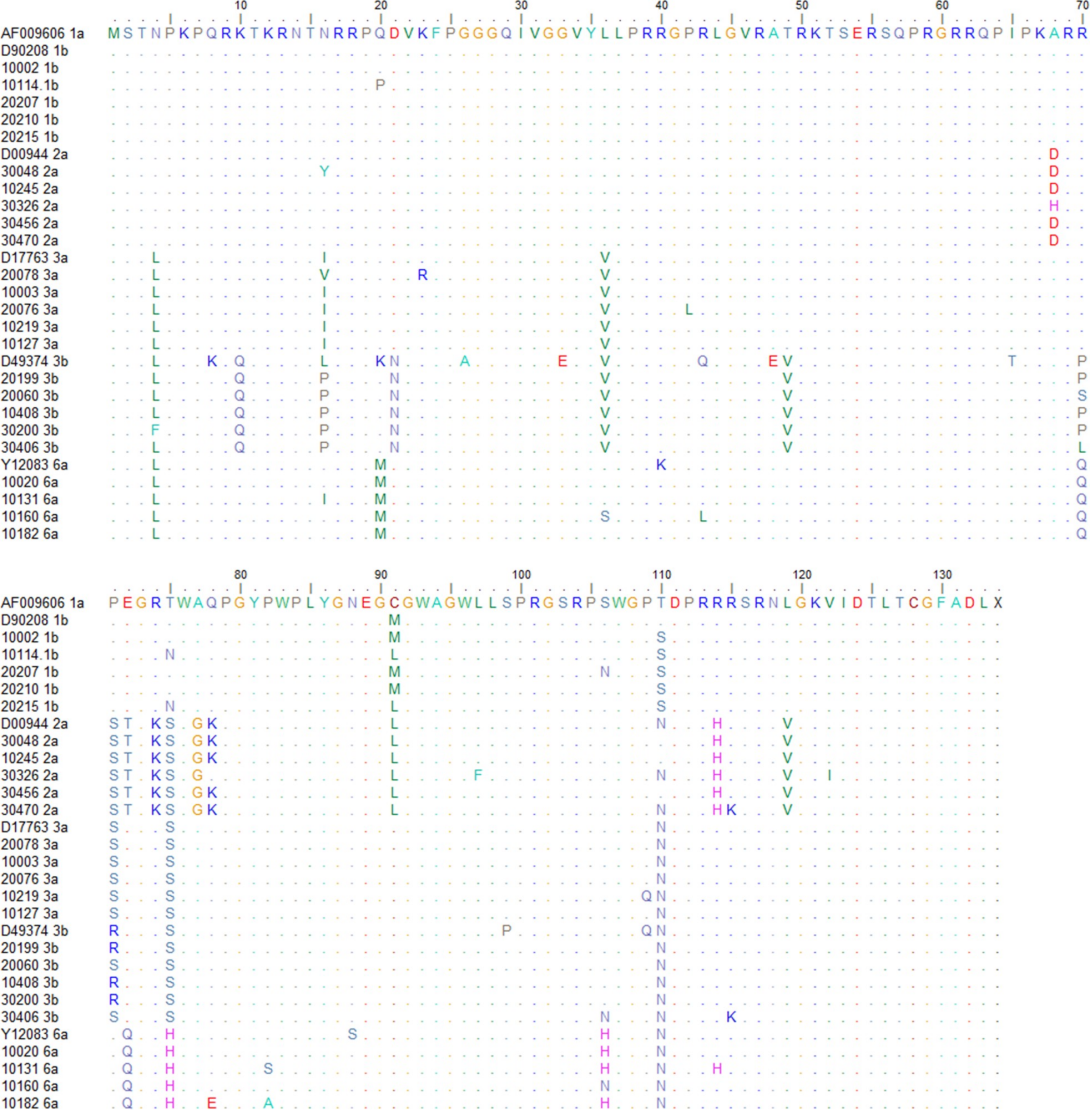
Amino acid sequences of core region among genotypes 1, 2, 3 and 6. The core sequence is numbered according to the genotype-1a strain with a GenBank accession number AF009606. Amino acids identical to those of the reference sequence are indicated by dots.

Molecular genotyping assay was performed for 363 samples with a positive anti-HCV result, and 110 samples yielded genotype and subtype results (Fig 2). For the remaining 253 samples, serotyping assay was performed, and 240 samples were successfully serotyped. Totally, 350 samples gave genotype results (Table 6). Sensitivity of the Fortune assay among genotypes 1, 2 and non-1/2 genotype was 93.39% (212/227), 91.55% (65/71) and 92.31% (48/52), respectively. No significant difference of the sensitivity of the Fortune assay was observed among the genotypes (P = 0.79). Moreover, three viremic subjects who failed to be diagnosed by the Fortune anti-HCV assay were infected by genotype-1 strains. Amino acid sequences of the core region from the representative stains with genotypes 1, 2, 3 and 6 were compared (Fig 3).

**Table 6:** Molecular and serological genotyping results (n = 363).

	Genotype 1	Genotype 2	Genotype 3	Genotype 6	Non-1/2 genotype	Untypable
**Molecular genotyping results**	70	23	13	4		
**Serotyping results**	157	48			35	13
**Total**	227	71	52			13

## Discussion

Theoretically, the Fortune POC oral anti-HCV assay has two advantages over the serum ELISA or chemiluminescence assay (CLA). It provides shorter turnaround time and higher sample availability. But it was not known whether these advantages were at the cost of sacrificing the clinical performance. Some POC assays lack sensitivity and specificity, which limits their application[22]. Therefore, we conducted this multicenter study to evaluate the performance of the new assay.

One of the major concerns for this colloidal gold assay is the specificity. The Fortune assay showed a high specificity (>97%) and a high PPV (>96%) in each Center. Ten cases gave a false positive oral anti-HCV result. Four had persistent HBV infection, four had primary biliary cirrhosis, and the remaining two had autoimmune hepatitis. It indicated that in rare cases, HBV related antibodies and autoantibodies might be the potential interfering substances leading to the false positive reactions. We further analyzed the specificity of the Fortune assay in testing the 77 apparently healthy subjects and 46 patients with non-liver related diseases. None of the 123 individuals were tested as oral anti-HCV positive. The results showed that the Fortune assay worked well either in patients with liver diseases or in individuals with low risk of HCV infection.

Another concern is its sensitivity. The sensitivity of the Fortune oral assay were 93.11%, lower than its specificity and PPV. Its CE-marked counterpart, the OraQuick assay, is reported to have a similar sensitivity of 94% in a recent meta-analysis [23]. Performance of the Fortune and the OraQuick oral assays were compared. The consistency was 96.35%. There were 10 discordant cases, among which nine were anti-HCV positive. The OraQuick assay was reported to have the highest sensitivity (98% vs. 88%) among the oral brands [23]. Our results demonstrated that the Fortune assay was even more sensitive than the OraQuick assay by identifying eight positive samples that could not be identified by the OraQuick assay; while the OraQuick assay detected only one positive sample than was undiagnosed by the Fortune assay. Furthermore, the OraQuick assay but not the Fortune assay failed to identify two viremic patients.

Sensitivity of the Fortune assay for diagnosing viremic patients were further assessed by using the archived HCV RNA data in the LIS. The assay could identify 97.46% of the viremic patients. In a previous study, the OraQuick assay showed a sensitivity of 88.52% when the serum anti-HCV assay was used as a reference; but its sensitivity rose up to 95.35% when the HCV viremia was added as the reference [24]. These results proved that the oral assays were more sensitive for identifying viremic patients. This is conceivable, because anti-HCV antibodies in the body fluids of an immunocompetent patient with active HCV infection are generally abundant enough to be detected[13]. Most of them are chronically infected for many years, and should be diagnosed and treated as soon as possible to prevent further progress of the liver impairment to liver cirrhosis and HCC.

However, three out of the 118 patients with active HCV infection were not diagnosed using the Fortune assay (Table 5), although all had high level of viremia and two of them has high serum anti-HCV titer. Moreover, other two cases with active HCV infection were not detected using the OraQuick assay (Table 4). The hook effect should be first considered, because the colloidal gold assays are both based on one-step homogeneous reaction, in which the hook effect may have an impact [25]. Therefore, 1:10 and 1:100 dilutions were performed for the three oral fluid samples with false-negative anti-HCV results generated by the Fortune assay. No results, however, turned to be positive. Thus, the hook effect was unlikely to impair the sensitivity of the oral assays, considering that the oral antibody concentration is only 1/800 of the serum antibody concentration [26]. There are four possible reasons for the relatively low sensitivity of the oral assays. First, the negative oral results might be attributable to the imbalanced distribution of the anti-HCV antibodies in human body fluids. Almost all of the immunocompetent viremic patients have high level of serum anti-HCV antibodies. However, a tiny minority might not have detectable oral antibodies accordingly. Second, there might be potential negative interference materials in the oral fluid. We did not collect the detailed data on the oral fluid sampling, but Lee et al. [27] reported that the presence of gingivitis, the use of dentures, and the consumption of tobacco and most types of food or drink did not affect the oral anti-HCV result. Future studies are needed to clarify the potential interference factors for the oral antibody assays. Third, the lower sensitivity might be attributable to the dilution of the oral fluid by the collection buffer [28]. Fourth, the binding of the anti-HCV antibodies with the coated HCV antigens in the kit might not be matched due to the variety of viral genotypes. For instance, our previous study indicated that genotype 3b had impact on the sensitivity of the Elecsys CLA anti-HCV assay [13]. Cha et al. [29] also found that the POC assays had shorter window period than the CLA assays when testing a genotype 3a seroconversion panel. But it was reported that the performances of POC assays was not likely to be related to HCV genotype [30]. In the present study, we compared the sensitivity of the Fortune assay among various genotypes and no significant difference was found. Sequence analysis demonstrated that the samples with genotype 3 harbored more amino acid polymorphisms in the first 50 amino acids of the core sequence than those with other genotypes, but it did not seem to impair the sensitivity. As we know, the Elecsys assay used a short peptide (from amino acid position 2 to 48) to capture the antibodies [19], while the Fortune assay included a much longer recombinant core protein (from position 1 to 130). This might partly explain why its sensitivity was not influenced by the genotype. Moreover, three viremic subjects missed by the Fortune assay were all infected by genotype-1 but not genotype-3 strains, and no amino acid polymorphisms were found. Consequently, sensitivity of the assay was genotype independent. From another perspective, the result revealed that the Fortune assay is pan-genotypically suitable for screening HCV infection. For some high-risk populations such as the IDUs and people receiving tattoos or body piercing, among whom genotype-3 or -6 HCV infection is endemic [19, 31], it is equally sensitive. Nevertheless, there were still 2 limitations on the genotype. First, NS3 and NS4 proteins were also used in anti-HCV assays. Their sequences among different genotypes were not compared in the present study, because NS3 and NS4 proteins are far less conserved than the core priotein [20],and the high sequence diversity will greatly increase the experimental complexity. Second, we failed to evaluate the sensitivity for genotypes 4 and 5, which are confined in several countries in the Middle East and Africa but seldom seen in the other regions around the world [1]. On the whole, further investigation is warranted to evaluate and improve the sensitivity.

What should be also emphasized, however, is that even the highly sensitive oral or serum anti-HCV assays have the risk of missing the immunocompromised patients or those with window-period HCV infection [32]. In our study, for example, one out of the three patients missed by both the Fortune and InTec assays was diagnosed as acute hepatitis C. The patient was from Center 1 (Table 1). Although their anti-HCV testing result were weakly positive or even negative (i.e. seronegative HCV infection [33]), they are suffering active infection. In these rare cases, despite the weakly positive or even negative serum or oral anti-HCV result, RNA or core antigen should be tested to confirm HCV infection [34].

On the other hand, either the ELISA or the much more sensitive CLA tends to yield weakly positive anti-HCV results when serum samples are tested. It often causes confusion to doctors and patients, especially where the HCV RNA test is not available or affordable [13, 35, 36]. For example, the weakly positive results with S/Co value ranging from 1.0 to 7.9 generated by the Vitros anti-HCV assay, and those with S/Co value ranging from 1.0 to 5.0 by the Architect anti-HCV assay, were unlikely to be true positives [36]. A “true” weakly positive result of an immunocompetent individual usually indicates that he experiences a spontaneous viral clearance or achieves SVR long before the anti-HCV antibodies disappear [21, 37]. Therefore, the application of the oral assays is still very cost-effective. It can help find out the vast majority of viremic patients in need of antiviral treatment, meanwhile for those with a weakly positive serum anti-HCV result, the unnecessary medical visits and psychological harm might be avoided [13].

In conclusion, the Fortune POC oral anti-HCV assay shows high specificity and PPV. The positive results it provides is of high diagnostic value. Although it seems more sensitive than the CE-marked OraQuick oral assay, the Fortune assay is less sensitive than the traditional serum counterparts. However, it is still sensitive enough for diagnosing viremic patients. Moreover, the assay is suitable for screening some high-risk populations such as the IDUs because of the satisfactory sensitivity for genotypes -3 or -6 patients. In the era of DAAs, it provides a rapid, completely non-invasive, and non-instrumental way for the accurate diagnosis of HCV infection. It is also suitable for the community-based testing services and home or supervised self-testing. With the implementation of universal HCV screening, more patients will be cured with the DAAs in the near future.

## Supporting information

S1 File Anti-HCV results (n = 1022) and HCV genotyping & serotyping results (n = 363). (XLSX)

S2 File Fortune anti-HCV results vs. HCV RNA results in 3 centers (n = 285). (XLSX)

S3 File HCV genotyping results, original Sanger sequencing results (n = 110). These results should be read by the Chromas, Bioedit or other software.
(ZIP)

## Abbreviations

DAAs: direct-acting antivirals
HCV: hepatitis C virus
POC: point-of-care
IDUs: intravenous drug users
CHC: chronic HCV infection
SVR: sustained virological response
PEG-IFN: pegylated interferon
NS: non-structural
ELISA: enzyme-linked immunosorbent assay
LIS: laboratory information system
S/Co: signal-to-cut-off ratio
LOD: limit of detection
PPV: positive predictive value
NPV: negative predictive value
CI: confidence intervals
CLA: chemiluminescence assay
